# Leadership styles in two Ghanaian hospitals in a challenging environment

**DOI:** 10.1093/heapol/czy038

**Published:** 2018-07-08

**Authors:** Matilda Aberese-Ako, Irene Akua Agyepong, Han van Dijk

**Affiliations:** 1 Institute of Health Research, University of Health and Allied Sciences, Ho, Volta Region, Ghana; 2 Research and Development Division, Ghana Health Service, Dodowa Health Research Centre, Dodowa, Shai Osudoku district, Greater Accra Region, Ghana; 3 Rural Development Sociology Group, Wageningen University, 6700 EW Wageningen, The Netherlands

**Keywords:** Context, management, leadership, capacity, motivation, frontline health worker, hospital managers, Ghana, low- and middle-income country

## Abstract

Hospital managers’ power to exercise effective leadership in daily management can affect quality of care directly as well as through effects on frontline workers’ motivation. This paper explores the influence of contextual factors on hospital managers’ leadership styles and the motivation of frontline workers providing maternal and new born care in two public district hospitals in Ghana. It draws on data from an ethnographic study that involved participant observation, conversations and in-depth interviews conducted over 20 months, with frontline health workers and managers. Qualitative analysis software Nvivo 11 was used to facilitate coding, and common patterns emerging from the codes were grouped into themes. Ethical clearance was obtained from the Ghana Health Service Ethical Review Committee. Contextual factors such as institutional rules and regulations and funding constrained managers’ power, and influenced leadership styles and responses to expressed and observed needs of frontline workers and clients. The contextual constraints on mangers’ responses were a source of demotivation to both managers and frontline workers, as it hampered quality health service provision. Knowing what to do, but sometimes constrained by context, managers described ‘feeling sick’ and frustrated. On the other hand in the instances where managers’ were able to get round the constraints and respond effectively to frontline health workers and clients’ needs, they felt encouraged and motivated to work harder. Effective district hospital management and leadership is influenced by contextual factors; and not just individual manager’s knowledge and skills. Interventions to strengthen management and leadership of public sector hospitals in low- and middle-income countries like Ghana need to consider context and not just individual managers’ skills and knowledge strengthening.

**Key Messages**

- Managers’ capacity to ensure effective leadership is highly dependent on contextual factors including national and health policies and directives. Effective decentralization will remain a mirage if public hospital managers are not provided with the necessary power to deal with the daily challenges that public hospitals face.
- Government needs to consider stepping up her responsibility to fully provide for the needs of health institutions if quality health care is to be realised.
- Ensuring frontline worker motivation to facilitate quality health care provision is dependent on managers being supported to meet frontline workers and hospitals’ needs.
- Frontline health worker motivation is closely tied to managers’ motivation. So facilitating managers’ work will not only motivate managers, but will contribute to motivating frontline workers as well.

## Introduction

Public sector district hospitals are often an essential part of primary health systems in low- and middle-income countries (LMICs) and avenues through which scarce national resources such as human, infrastructure, equipment, tools and supplies are channelled to provide primary referral care to the majority of the populace (Barasa *et al.* 2015; Martin *et al.* 2003; Mills 1990). Yet, there remain gaps in the literature about their management and leadership, how these affect worker motivation and thereby efficiency and quality of care.

Management and leadership are inter-related, but not completely identical concepts. Management has been defined as planning and using resources efficiently to produce intended results (Acquino 2015; Mansour *et al.* 2005). Common functions of management include to plan, organize, direct, control, coordinate, budget, innovate and represent the organization (Acquino 2015; Mansour *et al.* 2005). Leadership in health care has been defined as mobilising, influencing and communicating the organizational vision to inspire, motivate and empower others to work towards achieving this vision (Acquino 2015; Borkowski *et al.* 2011; Mansour *et al.* 2005). Effective leadership requires some managerial skills, and vice versa. Three leadership styles widely discussed in the literature are: transformational, transactional and laissez-faire (Antonakis *et al.* 2003; Bass 1997; Casida and Pinto-Zipp 2008).

Transformational leadership is able to guide subordinates to feel intrinsically motivated to perceive their performance in terms of the interest of the general good, so they strive to promote organizational goals (Antonakis 2001; Antonakis *et al.* 2003). Elements of transformational leadership include intellectual stimulation by encouraging worker creativity and innovation, encouragement and support through mentorship, inspirational motivation through presenting a clear vision, meaning of task, empowering and giving subordinates challenging tasks. Leaders also have charisma and serve as role models who take initiatives (Ahmad *et al.* 2013; Antonakis 2001; Antonakis *et al.* 2003; Bass 1990). Transformational leadership has been noted to be suitable for transforming individuals and the entire organization, which faces a dynamic evolving situation and requires learning to facilitate adoption, transformation of organizational culture and progress (Bucic *et al.* 2010; Borkowski *et al.* 2011). Transactional leadership is defined as leadership style that ensures that workers work according to the rules and regulations of the organization (Antonakis *et al.* 2003). Transactional leadership style punishes those who do not work hard and rewards those who are hard working. It also includes leaders who would wait till things go wrong, then they come in to punish, correct and teach, it is a form of carrot—stick approach to leadership (Antonakis 2001; Antonakis *et al.* 2003; Bass 1997). Bass (1990) contends that transactional leadership is more feasible to organizations in modern times in getting their staff to perform tasks for a fee. However, he argues that such an approach gives room to mediocrity, as it is ineffective and counterproductive, because staff might not take initiatives but will only wait for events to occur before they take action. Studies suggest that transactional leadership can only be exercised when the leader has power to reward and to punish, which is lacking in most public sector organizations (Bass 1990; Hsu *et al.* 2006). However, Lo in Ahmad *et al.* (2013) argues that transactional leadership has proven to be effective for change and efficiency compared to other leadership styles. Alimo-Metcalfe (1999) suggests that at a simplistic level, the distinction between transactional and transformational leadership marks the difference between just managing vs managing as well as leading. A study of nurses in Malaysia found that there was a strong relationship between job satisfaction and transformational leadership compared to transactional leadership (Ahmad *et al.* 2013).

Laissez-faire leadership style is viewed as leadership that does not make decisions to guide, correct or inspire subordinates to do their work (Antonakis 2001; Antonakis *et al.* 2003; Bass 1990; Bass 1997). This style concerns leadership that chooses not to apply the rules and so allows workers to do whatever they choose, even when they do not work in the interest of the organization. While Bass (1990) considers laissez-faire as one of the categories of leadership under transactional leadership, Barbuto (2005) reports that laissez-faire leadership is not included in the conceptualization of leadership, because it indicates the absence of leadership. Experiments on leadership in the boys scout in the USA and in a study by Katz, Macoby, Gurin and Floor reported in Barbuto (2005) of railroad section groups considered as unproductive, found that the leaders exhibited laissez-faire leadership styles, resulting in confusion, low productivity and poor work quality. Bass argues that transformational and transactional leadership are not static and a leader could practice the two concurrently, because transformational leadership style is an extension of transactional leadership (Bucic *et al.* 2010).

Leadership involves influence and influence draws on power. Power refers to the ‘… the capacity to effect (or affect) organizational outcomes’ (Mintzberg 1983)*.* Groups external to the organization including policy makers, pressure groups as well as internal groups such as managers, supervisors and workers have different forms and levels of power, which they exercise to influence organizational processes and outcomes (Mintzberg 1983; Pfeffer 1981). The actors in Management and Leadership are some of the most powerful actors within hospitals as they wield different forms of power such as reward, coercive, legitimate, referent (based on identification) and expert power, which enables them to influence organizational processes and outcomes (Mintzberg 1983). The extent of their power is however affected by contextual factors in the health sector such as organizational and institutional arrangements, national and sub-national policies, rules and regulations. Due to their expertise, skills and access to clients, health workers such as doctors, nurses and nurse-anaesthetists wield a lot of power (Lipsky 1980; Mintzberg 1983; Pfeffer 1981). This influences the extent of the power of the hospital management and leadership. Ortner (2006) has noted the omnipresence of power in social interactions and thus consciously and unconsciously power is enacted in interactions. In the case of the hospital, studies have noted that power determines how health managers organize health care provision in health facilities, how they interact with health workers, how workers interact and collaborate with colleagues as well as their interactions with clients (Aberese-Ako 2016; Pfeffer 1981, 1992). Studies report that where managers lack the capacity to exercise power to meet the personal and work needs of health workers, workers lose trust in managers, which demotivates workers and contributes to anti citizenship behaviours (Konovsky 2000). Workers have been noted to exhibit negative power by refusing to cooperate with colleagues to provide quality care, reporting to work late and absenteeism, which affects clinical decision making on patient care, thus compromising quality health care delivery (Aberese-Ako 2016; Coombs 2003; Fox 1974).

In resource poor countries such as Ghana, studies suggest that negative worker attitudes including absenteeism, conflicts, lateness to work, poor citizenship behaviour, which have a direct impact on quality of health care provision in public health facilities are largely an outcome of poor worker motivation (Aberese-Ako *et al.* 2015; Agyepong *et al.* 2004; Ansong-Tornui *et al.* 2007; Bosu *et al.* 2007; Dieleman *et al.* 2006; Mbindyo *et al.* 2009). Motivation refers to ‘…an individual’s degree of willingness to exert and maintain an effort towards organizational goals’ (Franco *et al.* 2004). Motivated workers exhibit positive attitudes that ensure that work is done efficiently and quality care provided to clients (Aberese-Ako *et al.* 2014; Aberese-Ako 2016; Adzei and Atinga 2012; Purohit and Bandyopadhyay 2014). Leadership style can influence job satisfaction and worker motivation (Bonenberger *et al.* 2014; Braun *et al.* 2013; Dieleman *et al.* 2006; Gilson 2003; Okello and Gilson 2015). Transactional leadership has been noted to motivate workers through an exchange process involving extrinsic rewards, rules and compliance (Caillier 2014). Transformational leadership on the other hand has been noted to motivate workers through inspiring them to rise above their personal interest for the good of the organization and its goals (Ahmad *et al.* 2013; Caillier 2014; Zhang and Bartol 2010).

In this paper, we explore how and why health system contextual factors affect leadership style and responses of hospital managers to frontline health workers and clients’ needs in two public sector district hospitals in Ghana; and the effects on frontline workers’ motivation to perform assigned tasks. We use the term ‘hospital manager’ to refer to senior health professionals who have been assigned the responsibility of leadership and management decision making and implementation oversight for frontline health workers to ensure the continued day to day functioning of the hospital.

Public sector hospitals in Ghana are led by a team of hospital managers. The head of the team is known as the Medical Superintendent. She/he is a medical doctor and works closely with the core hospital management team. The core hospital management team comprises four or five multi-disciplinary senior professionals namely: Medical Superintendent, Health Services Administrator, Accountant, Pharmacist and the Deputy Director of Nursing Services for the hospital. Beyond this core, the heads of the functional departments of the hospital namely the laboratory, pharmacy, maternity, outpatients, general, children and maternity wards are considered as members of a wider management team. Ward managers also known as in-charges are senior nurses, directly responsible for supervising nurses and supporting frontline health workers in the wards.

## Methods

This article draws on data from an ethnographic study in two highly urbanized community public sector hospitals, which we refer to as Grace and Adom hospitals in one region in Ghana. Adom, a specialist referral hospital, was selected to provide insights into the study question in the context of a bigger district hospital with specialized departments. Grace hospital provides basic maternity services and was chosen to help understand the same issues in a smaller district hospital without specialized departments. They were selected by an initial purposive stratification of the hospitals in the region into two groups according to size and complexity and a subsequent random balloting for one hospital in each group. The first author, M.A., collected data in the maternity and new-born units of Adom hospital from January to September 2012 and in Grace hospital from October to December 2012. In a second phase of the same study, she collected data in Grace hospital in July and August 2013 and in Adom hospital from October to December 2013. Follow-up interviews were carried out in Grace hospital in March 2014, and in Adom hospital in October 2014.

Participant observation was the core methodology for this study, supplemented with conversations and indepth interviews to check and triangulate the observations. Participant observation allowed first-hand experience of the researcher on daily hospital management and leadership practices and the interactions that produce (de)motivation. Employing interviews ensured that matters that were not immediately obvious were clarified and it also enabled an exploration of core issues in depth (Patton 2002; Yin 2009).

Interview respondents were purposively selected, because they had a specific role or relation to the events observed in the hospital. Face-to-face unstructured indepth interviews were conducted with hospital managers and frontline workers. A total of 134 conversations and 52 interviews were conducted with the different categories of study participants (Table 1). Grace hospital had no orderlies in the maternity ward, but ward aids did the same jobs as those referred to as orderlies in Adom hospital. This explains the absence of orderlies and a focus on ward aids in Grace hospital as reported in Table 1.

**Table 1.** Methods used to obtain data and categories of study participants

Category of workers	Data collection methods	
	Conversations	Indepth Interviews
**Adom hospital**		
Accounts officer	–	1
Hospital managers	3	4
Department managers	5	6
Ward managers	6	4
Nurses and midwives	62	12
Medical doctors	16	6
Medical doctors who left	1	2
Nurse-anaesthetists	3	3
Ward aids	2	2
Orderlies	3	3
**Grace hospital**		
Hospital managers	3	4
Department managers	2	3
Nurses and midwives	23	7
Nurse who left the facility	1	1
Ward aids	4	–

Conversations are defined as casual talk in everyday settings or a talk or spoken encounter (Goodwin and Heritage 1990). M.A. conversed with department and hospital managers, as well as the different categories of frontline health workers such as nurses, doctors, nurse-anaesthetists, accounts officers and ward aids. Conversations helped to gain a better understanding of management, leadership, power relations, organizational practices and interactions among the different actors.

Data were collected on managers’ decision making processes, leadership practices, administrative processes, communication and interaction between hospital and department managers, ward managers and frontline workers. Other areas of interest were worker attitudes, motivation and experiences. Additionally, documents such as health policies, guidelines and circulars were reviewed to enhance understanding of management and administrative decisions and practices.

Notes from observations of events, participation in meetings, workshops and conversations were jotted down in field notebooks. The notes were refined and expanded at the end of each field visit in line with standards in ethnographic studies (Emerson *et al.* 2005). Interviews were recorded by M.A. and transcribed verbatim by a research assistant. Observation notes, conversations and transcribed interviews were typed and transferred to Nvivo (version 11) software for qualitative analysis. A coding list on common themes that arose from the data was generated using the software. Subsequently, the data were systematically analysed to identify patterns, differences and contradictions.

Drawing on the literature, and focusing on the leadership functions of mobilizing, influencing, inspiring, motivating and empowering followers, we developed a simple guide for analysis of the data for evidence of transactional or transformational hospital manager leadership as in Table 2. Where managers avoided making decisions and trying to resolve problems, we classified the leadership style as laissez faire.

**Table 2.** Features, strategies and tactics used to guide analysis of hospital manager leadership behaviour as transactional or transformative

Leadership behaviour	Leadership style	
	Transactional	Transformational
Mobilizing	Hospital managers rely on ‘hard’ power, e.g. positional power to mobilize action	Hospital managers draw on ‘soft’ power (e.g. admiration, respect) as well as ‘hard’ power to mobilize action
Influencing	Carrot (rewards) and stick (sanctions) approaches are used to get followers to carry out roles and assignments. Reliance on ‘hard’ power	Shared meaning of a desirable future state is provided, followers helped to understand their contribution and challenged to use their roles and assignments to work towards it. Reliance on ‘soft’ as well as ‘hard’ power
Inspiring	Focus on specifying and communicating standards for compliance and what constitutes effective and ineffective performance and related rewards and sanctions	Involving followers in the development of shared vision of desired and attractive future states, communicating the vision, helping followers to understand their contribution to the shared vision and sharing credit for success
Motivating	Focus on contingent rewards and recognition	Attention is paid to individual as well as team needs for growth, and support provided to help team members attain their full potential
Empowering	Monitoring of followers is focused on individual errors and deviations	Followers are stimulated and encouraged to be innovative, creative and problem solving. Avoidance of individual public ridicule and humiliation for failures and recognition of system as well as individual causes of failure

Adapted from Avolio *et al.* (1999) and Bass *et al.* (2003).

Ethical clearance was obtained from the Ghana Health Service Ethical Review Board (approval number GHS-ERC: 06/01/12). Written and verbal informed consent was obtained from interview participants, and verbal consent to document information from conversations. In line with ethical principles, pseudonyms are used for hospital names and the exact location in Ghana is not disclosed.

## Limitation of the study

M.A.’s personal experiences became part of the data collection and analysis process, which introduced a personal element and some degree of subjectivity in the study. To minimize possible subjectivity bias, data from different sources were triangulated. Additionally, the data, analysis and conclusions were independently reviewed by the two co-authors. Finally, the analysis and conclusions were presented to the study participants for validation in preliminary dissemination forums.

## Results

**Figure 1.**
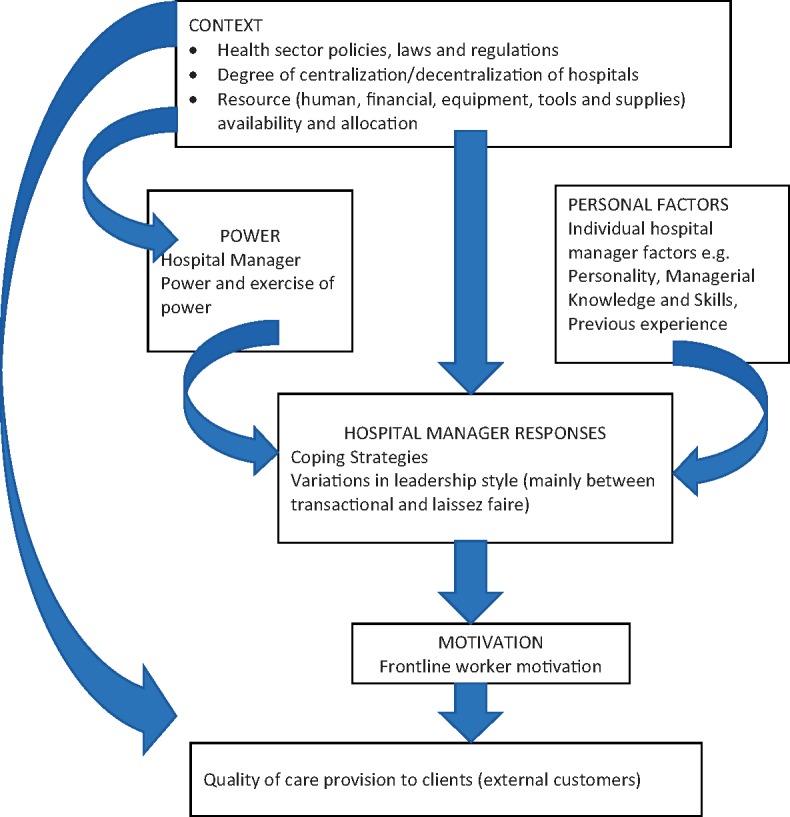
Contextual factors and hospital managers’ leadership style, decision making, responses and health worker motivation in two hospitals in Southern Ghana

Figure 1 summarizes the outcome of our analysis in the form of a framework of the effects of context on hospital managers’ power, leadership style and decision making responses to the needs of health workers (internal customers); and ultimate effects on motivation as well as the ability to provide effective and quality services to clients (external customers). We expand on this summary in the rest of our presentation of findings.

### Frontline workers (internal customers) motivation and quality of care and responsiveness to clients (external customers)

Frontline workers perceived that their managers were not responding promptly to their work needs such as essential drugs, medical supplies, equipment and infrastructure needed for quality service delivery. They interpreted this failure as due to hospital managers having other priorities. This made them feel frustrated and demotivated:

*Now all that they [hospital managers] are thinking of is where to save money and not staff interest. Look at the number of people squeezed over there, doctors, nurses and nurse-anaesthetists that is where we rest and eat. People are leaving here, because they are not happy, due to lack of job satisfaction.* ^1^

*Management is not looking at the needs of the hospital or the conditions under which staff are working. There are many challenges and constraints, some of which could have been resolved within, but there is a failure to resolve them, which is one of the major causes of our problems.* ^2^

*We are not asking for money, but when you come to work and there is no gauze, no linen, the doors are broken or no theatre doors, patients lying on the floor and no vital signs taken. These small small things to make your work comfortable*.^3^

*It (hospital) lacks a lot of things, so I am trying to go along with them. …it affects my motivation because things are not there to work with. Yesterday there were no cord clamps, so I had to go to one of the pharmacies out there.* ^4^

Some of the demotivated workers demonstrated their frustration and disappointment in their managers by exhibiting negative attitudes towards clients, such as shouting at clients and coming to work late; that reduced quality of care and responsiveness.^5^

### Context and hospital managers’ power

Managers admitted that some of the complaints from the frontline workers were true but explained that they were constrained by context. The requirements of the procurement law contributed to bureaucratic procedures that slowed down decision making and implementation of projects and the acquisition of equipment, tools and supplies, to meet workers and their hospitals’ needs. A manager cited his frustration over how the procurement process contributed to bureaucratic bottlenecks that constrained managers from taking decisions on the routine repairs of air conditioners:

*… we have planned to repair air conditioners. First, we have to ask for public bidding. They will submit their company proposals. We will do competitive bidding. We will select the one who qualifies and then we will give the contract. We will sign the contract, then they will come and do the servicing and now the last bit between when the thing is needed and when the thing is provided is a long duration of time. Although all the administrative processes are being taken care of—so administratively the institution is running perfectly, but managerially on the day to day basis the staff on the ground do not see it running perfectly. So I seem to feel that the perception they (workers) have (perception that managers are not showing leadership in responding to workers and the facility’s needs) is true. Even me, sometimes I get sick.* ^6^

The legislation that currently governs public sector service delivery in Ghana is the Ghana Health Service and Teaching Hospitals Act 525 of 1996 (Republic of Ghana Act 525, 1996). Under Act 525, the health sector is organized in an agency model with provider, purchaser, regulatory and training agencies under the oversight and coordination of the Ministry of Health (MOH). The Ghana Health Service (GHS) is the general public sector health service delivery agency.

The GHS has a hierarchical structure with a governing council and a Chief Executive known as the Director General. Under the Director General are 10 regional health directors presiding over service delivery in the 10 regional health directorates. Under each regional health director are district directors who preside over district level service delivery. District hospitals are part of the primary care system and the first level of referral (Agyepong 1999; Larbi 1998).

Act 525 gives hospital managers the power to collect and retain 100% of user fees known as internally generated funds (IGFs) to ensure efficient and quality services. However, the power to employ professional staff and the human resource budget remain centralized at national level and outside the jurisdiction of hospital managers. The human resource division (HRD) of the GHS at national level recruits and posts professional staff to the regional health directorates. The regional health directorates in turn distribute the staff to the districts and facilities within the region. Hospital managers request staff from the regional health directorate, who in turn request staff from the HRD at national level. A request does not automatically guarantee that the calibre and numbers of professional staff requested will be posted to the requesting region or facility. Human resource availability and government’s ability to pay (authorization from the Ministry of Finance) influences the response.

Another legislation that affects hospital managers’ decision making is the public procurement Act 663 (Ameyaw *et al.* 2012). The Act requires that a procurement team should be formed in each government facility. The team meets every quarter to review procurement requests and procedures, and review supplier bids for items needed to be procured based on clearly agreed and documented criteria in relation to the items being procured. The same procurement procedure, which applies to the ministries such as where no lives are at stake or medical emergencies in question, applies to the Ghana Health Service’s health facilities, where lengthy procedures can sometimes make a difference to the timely availability of life-saving commodities.

Hospital managers’ power to address needs for infrastructure, equipment, tools, medicines and other supplies was also constrained by other factors. These included longstanding inadequate central government subventions for recurrent expenditure,^7^ as well as capital investments for infrastructural and resource uncertainty due to erratic and unpredictable reimbursement from the National Health Insurance scheme.^8^^,^^9^

### Hospital managers’ responses

In response to their contextual constraints hospital managers adopted a variety of coping strategies often within a transactional leadership style. However, they also moved between leadership styles. Depending on the nature and the amount of power available within the constraints of context, laissez faire was observed. We also observed some (but fewer) instances of transformational leadership.

### Coping strategies within a transactional leadership style

Coping strategies included the identification and use of windows of opportunity; skilful networking, lobbying and collaboration; flexible interpretation of rules; abandoning failing strategies to try alternatives and shaming higher authorities into action, and appeals to higher levels for reversal of unfavourable decisions.

Managers in both hospitals described how they took advantage of the window of opportunity presented by an impending peer review exercise from the regional level to sidestep the normal lengthy bureaucratic procurement process and quickly acquire much needed hospital equipment, supplies and to renovate existing infrastructure.^10^ By taking advantage of the special circumstances of the peer review, they were able to avoid being accused of flouting procurement laws, or being seen to break managerial and administrative rules.

Managers in both hospitals also described using networking, lobbying and collaborating with international donors to enable much needed infrastructure development in the form of additional ANC consulting rooms.^11^ They also stretched and gave fairly elastic interpretations to the use of IGFs and other resources for recurrent expenditure. Adom hospital managers used IGF and financial support from the regional health directorate to renovate the maternity theatre, which was in a deplorable state.^12^^,^^13^ Grace hospital managers used IGF to construct a new administrative block and to renovate and convert a structure previously occupied by the administration into a maternity block, to enable provision of comprehensive maternal and new-born care.^14^

Adom hospital experienced a water shortage crisis, which hampered quality service provision especially of surgical care and related infection prevention and control. The situation greatly frustrated and demotivated workers, especially the theatre staff with frontline health workers blaming the crisis on failed leadership from hospital management. Negotiation with the Ghana water company and the MOH to address the crises by supplying water from the mains was repeatedly unsuccessful. They changed strategy and successfully lobbied government to construct bore holes for the hospital; and additionally contracted with a private water distributor to supply the hospital theatre and wards with water to augment the supply from the borehole.^15^ This enabled workers to provide uninterrupted theatre services to the clients, which was a source of relief to the workers. A senior doctor who had been frustrated with management’s response to their work needs expressed joy and satisfaction when on one occasion she had water, linen, gauze and house officers to work with.^16^

Trying to shame authorities into action was described as a last resort in one case. The shortage of health workers especially medical doctors and nurse-anaesthetists providing maternity services had resulted in a situation in Adom hospital where the few doctors at post felt overworked, frustrated and demotivated. This in its turn was contributing to a vicious cycle of more doctors leaving and workloads rising further.^17^ The general feeling was that hospital managers and higher level authorities were not exercising effective leadership to address the problem and facilitate quality care.^18^^,^^19^ Hospital managers had repeatedly and unsuccessfully tried to address the problem using the normal channel of requesting for doctors from the regional health directorate. Additionally, they had applied strategies such as lobbying higher authorities and encouraging and negotiating with individual doctors to apply to work in the hospital. Hospital managers also tried to resolve the problems by going beyond the officially approved incentives to provide unapproved monetary incentives for doctors, as a strategy to retain them.^20^ Finally in frustration and desperation; and to strategically compel the regional and national levels to address the shortage of doctors the hospital posted a public notice indicating that they were no longer able to provide maternity services for lack of doctors. It yielded some but not the full desired response, probably because the higher levels were themselves constrained by the absolute national shortages. Two doctors were posted, though in relation to workloads, the department estimated it needed more like 25 doctors to operate optimally.^21^

In an appeal to a higher level to reverse an unfavourable decision, Grace hospital wrote to the regional health directorate to rescind a decision to withdraw the only doctor in the maternity from the hospital. The decision had resulted in a protest from the nurses in the maternity. The reason the regional directorate gave for withdrawing the doctor was that the facility had failed to develop the facilities for emergency obstetric care to enable the doctor to fully utilize his expertise. His retention in that facility was a waste of scarce expertise.^22^ Additional to appealing the decision, the hospital also quickly devoted most of its limited resources to complete the maternity complex and emergency obstetric care facilities, so that they could retain him.^23^

### Laissez-faire leadership style

Much of the laissez-faire leadership style observed appeared to be driven by a sense of powerlessness and was often related to the issue of human resources. For example, despite the various creative strategies adopted by managers in both hospitals they could not get the required number of doctors needed in relation to workloads. The few doctors available regularly complained of being overworked. A hospital manager in Adom hospital observed:

*Everybody [doctors] is busy, even doing more than the regular hours. So having somebody to cover the weekend is almost like slaving somebody to do it. We have doctors who are falling sick on the job*.^24^

Faced with a sense of powerlessness, hospital managers sometimes resorted to laissez-faire leadership style, and looked the other way when work was still left undone, because they did not want to push doctors beyond their capabilities.

The inconvenience and time required to pursue the long bureaucratic processes to discipline a worker who repeatedly committed offences, including the referral to the higher levels with the power to hire and fire; as well as the doubtful outcome of the process appeared to discourage managers from making the effort. At the simplest, the procedure required first a verbal query, followed by two written queries and to finally write a report to the regional health administration. Appeals and reviews could mean further investigation and hearing of the case at national level.^25^ Moreover, getting rid of staff could increase workload since managers could request for the transfer of a staff from the facility, but this did not guarantee a replacement from the regional health directorate. A department manager summed up how managers involuntarily succumbed to laissez-faire leadership due to a lack of power to demand accountability from subordinates as follows: ‘You just depend on the goodwill of the doctor…. Some of them you give them a query and they even tell you they forgot to answer it’.^26^

There was an annual staff performance appraisal system, but the data did not appear to be used at the central level where decisions were taken. Promotion and salary were based on years of service and not on performance, which made it impossible for managers to hold staff accountable. In the words of one hospital manager:

*… people are promoted like sheep. When you are due for promotion, you are promoted and that is all. It does not matter whether you are doing the work or not. You can fill in the appraisal forms and indicate all that you want about the doctor, but the fellow will be promoted.* ^27^

Another hospital manager suggested the human resource management problems where: ‘People know that they cannot be sanctioned; they cannot be punished and cannot be sacked’^28^ were peculiar to the public sector, which was unlike the private sector.

The effect of the centralized human resource management system was probably worsened by observations that hospital managers appeared to depend more on transactional leadership styles. The ‘soft’ transformational leadership skills of encouragement and support through mentorship, inspirational motivation through presenting a clear vision, meaning of task, and empowering subordinates etcetera could perhaps have provided a little more leverage over staff behaviour and motivation despite the context of the centralization of the power to hire and fire. The results of the large amount of laissez-faire leadership appeared to be negative.

Sometimes there was poor collaboration between professionals from the different departments. This sometimes resulted in absenteeism, or some workers closing early and leaving the work to those who chose to stay, fuelling conflicts between workers, frustration and demotivation. The effects could further worsen some of the human resource crisis the hospital was already facing. An example was a doctor who was described as very hardworking, but eventually left Adom hospital. On enquiry, he explained that he left because his colleagues manipulated the system, by doing the easy surgery cases and leaving the difficult cases for him and he eventually got overwhelmed with work. He admitted that on hindsight he should have complained to his head of department, who failed to notice the problem, which could have probably been averted by redistributing tasks.^29^

The frustration was shared by frontline workers as well as hospital managers. For instance a department manager expressed frustration and helplessness over a nurse-anaesthetist who could disappear for hours when on schedule to work in a private hospital. The manager felt they had no power to change the situation and had decided to just accept sharing the anaesthetist with private hospitals, even when he was scheduled to work in the hospital.^30^ This use by professional staff of part of their official work hours to practice *locum* was a particular challenge for hospital managers and strong opinions were expressed on the issues despite handling it with a laissez faire approach because of a sense of powerlessness. Most of the time they tried to use transactional leadership styles such as cautioning workers, followed by threats before falling back on laissez-faire. An Adom hospital manager admitted that she had had several confrontations with a doctor, due to his frequent absenteeism from his official work to practice *locum*.^31^ A hospital manager in Grace hospital who had resigned herself to laissez-faire leadership after having lost the battle over a doctor’s *locum* practice commented with frustration: ‘You cannot serve two masters and be faithful to both’.^32^ Some of the nurse managers frowned on nurses who shuttled between the hospital and *locum* practice, but chose not to report to a higher authority, while others threatened to report such subordinates to hospital managers.^33^ A hospital manager bitterly attributed perceived poor quality care in public hospitals compared to private hospitals, to *locum* practice:

*You will not hear of significant maternal deaths [in private hospitals], because all the professionals are there. They do not sit at their posts in the main hospital. They go there in the evenings or afternoons to do the cases and they get good results. But the public sector, which is meant for everybody who cannot afford, is left to hang by itself.* ^34^

### Transformational leadership

There were some examples of transformational leadership. Hospital managers sought to inspire and empower staff by promoting further development in critical professional areas such as in anaesthesia. They were successful in using some of the IGF to fund further training of some nurses, to become nurse-anaesthetists and retaining them afterwards. The strategy helped to inspire some young nurses to improve their skills by training to become nurse-anaesthetists to boost the number of nurse-anaesthetists in Adom hospital. Some of such trained nurse-anaesthetists said in conversations that they were intrinsically motivated to work as nurse-anaesthetist, as this gave them some status and also empowered them to work without having to answer to doctors unlike nurses.^35^

Some of the efforts were foiled by frontline worker perceptions of hospital manager unfairness in some procedures. Nurse-anaesthetists frequently complained that hospital managers were favouring medical doctors by creating incentive packages for them,^36^ so they felt demotivated and some exhibited negative attitudes including absenteeism, lateness to work, sacrificing some of their official time for *locum* practice and poor cooperation with nurses and doctors on surgical cases.^37^ Nurses in some of the wards also complained of discrimination, because some wards were provided with lunch and others were not. Managers explained that there were conflicting directives from the GHS and the MOH on the provision of incentives to workers. On one occasion managers received a directive from the GHS indicating that the Ministry of Finance and Economic Planning had ordered that the IGF should not be used to provide financial incentives to workers. Also managers were asked to justify incentives for staff.^38^ On the other hand, managers were told at managers’ meeting by their superiors from the GHS that they should offer staff incentives that were within their means.^39^ Managers addressed the conflicting directives by writing to the GHS to justify incentives for doctors, which was approved. A similar request for incentives was made for the other professional categories, but the GHS had not responded to the request three years after it had been made.^40^ So sometimes managers made *ad hoc* decisions on incentives for staff, which exposed their vulnerability and gave room for the different professional groups to lobby for incentives. A powerful and influential group like the doctors had some of their demands met.^41^

This study did not investigate individual hospital manager factors. However, we have noted it in the summary framework because the observations and data suggest that in the area of transformational leadership, quite apart from any contextual constraints, limits of individual hospital manager experiences, knowledge and skills may have influenced its relatively limited use.

## Discussion

Hospital managers in this study adopted several coping strategies within a predominantly transactional leadership style to deal with the contextual constraints. They however sometimes adopted laissez-faire style and in a few instances also transformational leadership styles, adapting their leadership style to the nature of the challenge. In the areas where they had moderate power over the issues, managers employed predominantly transactional leadership and occasionally transformational leadership. On the other hand, they resorted to laissez-faire leadership when they felt most powerless. This was especially in relation to staff discipline and accountability issues where the centralization of the power to hire and fire severely limited the ability to exercise transactional leadership.

Managers’ expressed that their inability to address all the needs of workers and their facilities as well as to institute appropriate disciplinary measures made them ‘feel sick’, frustrated and demotivated. Frontline workers also felt demotivated when managers were not able to meet their personal and work needs, which in turn affected their responsiveness and quality of health care provided to clients. On the other hand, managers always expressed feelings of satisfaction and motivation, whenever they were able to meet some of their workers and hospitals’ needs. Sometimes workers also appreciated managers’ efforts but at the same time they felt that there was more potential in their hospitals, which their managers were not exploiting to meet their needs.

Managers on the other hand felt that they were doing their best in the face of contextual constraints on their power to address issues such as national policies, directives and processes. Within their predominantly transactional leadership style, hospital managers adopted various coping strategies to deal with contextual challenges where they saw the space to do so. Laissez-faire leadership while appearing to be a form of a lack of power to make decisions, can also in itself serve as power, for in this study it demonstrated that the decision to refuse to exercise power or to take an initiative in itself influenced organizational outcomes. The consequences of exercising power this way appeared to be on the whole negative.

Managers demonstrated skilfulness in the use of coping behaviour to rise above administrative bottlenecks and bureaucracy to deal with policy and contextual constraints that gave them little power to make decisions on critical resources aimed at meeting their facilities’ needs. Similarly, Pfeffer's (1981) study of power in organizations suggests that the professional manager is expected to operate in a more bureaucratic environment, where she has less control and ownership over resources. Mintzberg’s study of management (in Daire and Gilson 2014) further indicates that for managers to be able to manage organizations they have to navigate in dealing with established procedures and management systems.

Erratic and unreliable sources of funding, which constrained hospital managers’ capacity to practice transactional leadership in meeting the personal and work needs of workers, made managers to ‘feel sick’, disempowered and frustrated. Other research work in a public teaching hospital in Ghana corroborates the finding that public hospitals in Ghana are unable to meet frontline workers and their facilities’ needs (Bohmig 2010; Böhmig 2010).

Policy and contextual factors weakened managers’ power to demand accountability from health workers, but at the same time they gave frontline workers a lot of power, which some of the workers abused. Lewis and Pettersson (2009) reveal that physicians and nurses hired, paid and deployed by ministries of health become accountable to central government and not to their facility managers. Consequently managers are not able to effectively supervise and discipline workers, who know that they cannot be sacked, so they are free to exhibit negative attitudes, which results in poor response to patient care. Other studies suggest that the social life of the organization is such that there is both hierarchical and vertical power, where professional workers especially nurses and doctors derive immense power from their skills, access to clients and the discretionary nature of their work (Lipsky 1980; Mintzberg 1979; Mintzberg 1983; Pfeffer 1981, 1992). So mangers need to make an extra effort to exercise transformational leadership such as getting workers to perceive a common vision and purpose (Ahmad *et al.* 2013; Borkowski *et al.* 2011; Bucic *et al.* 2010). This will ensure that workers exercise their power positively to bring the different professional groups on board to work in the interest of achieving organizational goals (Aberese-Ako 2016; Lipsky 1980; Pfeffer 1981, 1992).

Mangers’ lack of power to exercise transactional leadership by effectively disciplining recalcitrant and corrupt workers confirms Bass (1990) and Huss *et al.*'s (2011), assertion that public sector leaders are not able to exercise transactional leadership to punish and reward workers, because they lack power. Similarly, Cornwall *et al.* (2000) suggest that the limited number of skilled workers and the difficulty in replacing them renders them immune to disciplinary actions. Braithwaite and Travaglia (2008) add that due to the shortage of staff, it is difficult to know how far to push accountability, for if clinicians are pressed too hard, they may leave or go into private practice. Huss *et al.* (2011) and Lewis and Pettersson (2009) report that corruption is one of the challenges that health managers have to deal with to ensure effective decentralization and effectiveness in hospital management.

The role of departmental managers in ensuring that power and leadership especially in health worker supervision is decentralized to the work floor level continues to remain a challenge as a result of the imbalance of power among the different health professionals. Similarly Daire and Gilson's (2014) study of nurse-managers found that there was professional power imbalance in favour of doctors. Nurse-managers felt disempowered, as they could not discipline doctors, who did what they pleased, because they were perceived to have higher ranks compared to the nurse managers. Also managers felt disempowered in the long process required to discipline a staff, as one had to depend on a manager from a higher level, who was not privy to the offence (Daire and Gilson 2014).

Managers’ expression of frustration and demotivation was an indication of frustration over the lack of power to exercise leadership at every level of the hospital. Managers felt demotivated because they could not exercise adequate leadership to deal with organizational and worker issues. Workers, on the other hand, were demotivated for lack of resources to work with, which limited their power to exercise their professional skills in the provision of health care. Similar studies on South African health facility managers suggest that extreme bureaucratic procurement process and the lack of power to make critical decisions on human resources frustrate and stress them (Holdt and Murphy 2007). Other studies on worker motivation equally reveal that lack of resources to work with and staff shortages contribute to demotivation, which could result in absenteeism and worker attrition (Aberese-Ako 2016; Adzei and Atinga 2012; Agyepong *et al.* 2004; Franco *et al.* 2004; Friedman 2005; Mbindyo *et al.* 2009; Rowe *et al.* 2005).

Hospital managers derived satisfaction, pride and motivation whenever they were able to exercise transformational and transactional leadership by using the IGF to address some of the needs of workers and their facilities. Motivation served as an end in itself because managers felt good and were further inspired to work harder to realise organizational goals. Similarly, Leon *et al.* (2001) report that facility managers in South Africa were motivated to work harder, when they were able to initiate or were successful in overseeing physical infrastructure development.

Effective leadership motivated some of the health workers, which resulted in workers exercising power positively by aspiring to achieve the hospitals’ vision of providing quality health care to clients. Motivated workers exercised power positively by exhibiting positive attitudes in client care, which contributed to quality health care provision. Laissez-faire leadership on the other hand sometimes contributed to demotivation of workers, who exhibited negative attitudes towards clients, which contributed to low-quality health care provision that resulted in the hospitals being given bad names. Other studies have found that effective human resource management and leadership contributes to worker motivation and performance and the contrary is true (Choi *et al.* 2016; Dieleman *et al.* 2009; Rowe *et al.* 2005).

## Conclusion

Power is core to determining the style of leadership that managers can exercise in public health care institutions in Ghana and their effectiveness. For managers to be able to exercise effective leadership in order to address workers and hospitals’ needs, which could boost frontline workers’ motivation for quality health care delivery, the following recommendations have been suggested. National and health policies should aim at giving hospital managers more power and autonomy in making decisions on critical resources including the right workforce mix, infrastructure development, drugs, medical supplies and equipment. This could facilitate timely planning and coordination of activities required for effective health care delivery. Adequate resources and financial support should be provided to boost managers’ capacity to meet workers and hospitals’ needs. There is the need for reforms in supervision and guidelines for assessing health staff for remuneration and promotion purposes within the public health care sector. Additionally, it will be important to build hospital managers’ capacity to exercise more transformational leadership styles.

## Footnotes

1 Conversation with a nurse-anaesthetist, 5 July 2012.

2 Conversation with a medical doctor, 18 July 2012.

3 Comments from a nurse-anaesthetist at a training workshop for health workers, Adom Hospital, observation notes, 24 May 2012.

4 Interview with a nurse manager, 30 July 2013.

5 Observation notes, 21 August 2012.

6 Interview with a hospital manager, 31 July 2012.

7 Interview with a hospital manager, 5 March 2014.

8 Interview with a hospital manager, 4 December 2013.

9 About half of both hospitals’ clients were NHIS subscribers and since the scheme sometimes delayed for over 6 months before reimbursing the facilities.

10 Interview with a hospital manager, 31 July 2012 and interview with two hospital managers, 6 August 2013.

11 Interview with a doctor, 29 July 2013 and conversation with a ward manager, 26 November 2013.

12 Observation notes, 30 April 2012 and interview with a former hospital administrator, 7 August 2013.

13 Interview with two hospital managers, 3 December 2013 and 31 July 2012.

14 Interview with two hospital managers, 6 July 2013.

15 Interview with a hospital manager, 4 December 2013.

16 Observation notes, 29 November 2017, Adom hospital.

17 Interview with two doctors who left Adom hospital, 4 August 2013; 8 September 2013; Conversation with a doctor who left Adom hospital for private practice.

18 Observation notes, 30 April 2012, Adom Hospital.

19 Interview with a doctor who left Adom hospital, 4 July 2013.

20 Interview with a hospital manager, 4 December 2013 and interview with an accounts officer, 17 August 2012.

21 Conversation with a department manager, 30 April 2012.

22 Interview with a hospital manager, 5 March 2014; official correspondence.

23 Interview with hospital manager, 5 March 2017.

24 Interview with a hospital manager, 31 July 2012.

25 Interview with a hospital manager, 5 June 2014, Grace hospital, Ghana Health Service (2003). *Code of Ethics and Disciplinary Procedures*, Accra, The City Printers.

26 Conversation with a department manager, 17 August 2012.

27 Conversation with a department manager, 17 August 2012.

28 Interview with a hospital manager, 31 July 2012.

29 Interview with a doctor who left Adom hospital, 4 August 2013.

30 Conversation with a doctor-department manager, 17 July 2012.

31 Interview with a hospital manager, 4 December 2013.

32 Interview with a hospital manager, 4 March 2014.

33 Interview with a hospital administrator, 29 November 2013.

34 Interview with a hospital manager, 31 July 2012.

35 Conversation with an anaesthetist, 5 July 2012.

36 Conversation with head of nurse-anaesthetists, 5 July 2012.

37 Conversations with head of anaesthesia, 5 July 2012.

38 Interview with a hospital manager, 4 December 2013; conversation with a department manager, conversations with nurses.

39 Interview with a hospital manager, 4 December 2013.

40 Interview with a hospital manager, 4 December 2013.

41 Interview with a hospital manager, 4 December 2013.
